# Effects of insecure attachment on fMRI resting state functional connectivity in poly drug use disorder

**DOI:** 10.1371/journal.pone.0318505

**Published:** 2025-02-12

**Authors:** Jürgen Fuchshuber, Karl Koschutnig, Andreas Fink, Johanna Alexopoulos, Henriette Löffler-Stastka, Human-Friedrich Unterrainer

**Affiliations:** 1 Department of Psychoanalysis and Psychotherapy, Medical University Vienna, Vienna, Austria; 2 Comprehensive Center for Clinical Neurosciences and Mental Health, Medical University Vienna, Vienna, Austria; 3 Center for Integrative Addiction Research (CIAR), Grüner Kreis Society, Vienna, Austria; 4 Institute of Psychology, University of Graz, Graz, Austria; 5 Department of Religious Studies, University of Vienna, Vienna, Austria; 6 Faculty of Psychotherapy Science, Sigmund Freud University, Vienna, Austria; 7 University Clinic for Psychiatry and Psychotherapeutic Medicine, Medical University Graz, Graz, Austria; Museo Storico della Fisica e Centro Studi e Ricerche Enrico Fermi, ITALY

## Abstract

**Background:**

Insecure adult attachment has previously been linked to more severe psychopathology and to alterations within neuronal connectivity on a structural as well as functional level. Little is known about the resting state functional connectivity (rs-FC) of the attachment system in patients suffering from poly-drug use disorder (PUD).

**Methods:**

The present study investigated rs-FC at two measuring points (t1: ROI-to-ROI; t2: seed-to-voxel) in a sample of PUD patients (n = 33; Age: M = 30y; SD = 8y; Female = 15%). Adult attachment was measured with the German version of the Experiences in Close Relationships Scale (ECR-RD8). Furthermore, insecure attachment was correlated with depressive symptoms (ADS), trait anxiety (STAI) and general psychopathology (BSI-53).

**Results:**

More insecure attachment was associated with increased trait anxiety, depressive and general psychiatric symptom burden in patients. Furthermore, we observed time-stable links between insecure adult attachment and increased rs-FC between the left lateral parietal default mode network (DMN LP) and bilateral parts of the salience network, as well as decreased rs-FC between DMN LP and medial parts of the DMN.

**Discussion:**

Implications of the present study are highlighting the association between attachment security and brain areas related to affect regulation.

## Introduction

Attachment theory has become an increasingly influential concept in psychological and psychotherapeutic research. Past investigations have convincingly demonstrated that adult attachment patterns are linked not only to differences in interpersonal behavior but also to affect regulation and a variety of psychopathologies. Specifically, insecure attachment was repeatedly shown to be associated with substance use disorders [1]. Correspondingly, chronic substance use is often conceptualized as chemical coping strategy directed at managing overwhelming negative affects [2,3]. This, however, leads to a vicious circle of escalating substance use and progressively impaired affect regulation capacities [4–6].

Expanding upon the seminal work of Ainsworth [7], besides secure attachment, insecure attachment styles can be sorted into three distinct categories: anxious-ambivalent, anxious-avoidant, and disorganized attachment patterns. Notably, these categories align closely with Bartholomew and Horowitz’s [8] taxonomy of attachment patterns in adults, which encompasses the anxious-preoccupied, dismissive-avoidant, and fearful-avoidant patterns. Brennan, Clark and Shaver [9] employed item-response and factor analytic methodologies to examine various adult attachment measurements comprehensively. Their investigation identified two overarching factors, known as *Anxiety* and *Avoidance*, which serve to encapsulate a significant portion of the multifaceted attachment construct. Attachment anxiety, one of these critical factors, is characterized by a pronounced association with the fear of rejection and an excessive need for attention. Individuals with high levels of attachment anxiety tend to experience a progressive elevation of stress when their partner appears indifferent or unavailable, a dynamic aptly referred to as hyperactivation. In contrast, attachment avoidance, the second pivotal factor, manifests as the deactivation of the attachment system, reflecting an inclination to obviate interpersonal dependency and intimacy [10]. The fearful-avoidant attachment pattern corresponds to high expressions of both anxious and avoidant attachment traits. In relation to either anxious or avoidant organized attachment patterns, this disorganized style is linked to more severe difficulties in affect regulation, interpersonal problems and psychopathological symptoms [11–16], as fearful-avoidant individuals want closeness to attachment figures and are unable to trust and rely on them, simultaneously. This contradiction poses a significant threat for a collapse of social coping strategies under intensely stressful conditions [17]. Previous research suggested that this style is prevalent in patients diagnosed with severe addictive disorders, such as opioid dependence or polysubstance use disorders (Schindler et al., 2019).

Recent decades have seen an increasing interest in the neurobiological underpinnings of the human attachment system. Neurochemically, attachment is primarily mediated by a complex interplay between dopamine, oxytocin, vasopressin and endogenous opioids [18–21]. Anatomically, Vrticka and Vuilleumier [22] proposed a network consisting of the ventral tegmental area (VTA), pituitary/hypothalamus, striatum, and ventral medial orbitofrontal cortex (OFC), amygdala, hippocampus, insula, anterior cingulate cortex (ACC), and anterior temporal pole (ATP) as crucially involved in social approach and aversion behavior. Furthermore, given its central role in (mental) pain processing and its high density of opioid receptors [23], the periaqueductal gray (PAG) was repeatedly suggested as a neurobiological node for the attachment system [24–27].

In recent years, a number of studies investigated the effects of attachment security on resting state functional connectivity (rs-FC) in the general population [28–30]. Rs-FC describes the temporal correlation between spatially remote neurophysiological events like BOLD signal fluctuations [31]. Rigon, Duff and Voss [28] observed stronger rs-FC between the bilateral amygdala and medial prefrontal regions associated with increased avoidance, while lower anxiety was associated with stronger rs-FC between the bilateral amygdala and bilateral caudate, thalamus and putamen. After adjusting for general psychopathology, van Hoof et al. [30] observed stronger functional connectivity between the left amygdala and the left lateral occipital cortex, precuneus, and superior parietal lobule related to a disorganized attachment. Moreover, disorganized attachment was associated with a decreased rs-FC between the left amygdala and medial frontal cortex. Finally, Deng, Zhang and Gao [29] reported positive correlations between avoidance and rs-FC between bilateral inferior temporal gyrus, bilateral orbitofrontal cortex, right postcentral gyrus as well as the frontotemporal network, while anxiety was associated with increased rs-FC between right PCC and the fusiform gyrus.

The research on the rs-FC characteristics of adult attachment is still in its beginnings and hitherto produced rather heterogeneous results. Yet, in general findings point towards a pattern of networks involving regions functionally connected to affect- and self-perception, affect regulation, as well as (social) approach and aversion behavior.

### Study aims

The present study aims to investigate the effect of insecure attachment styles on functional connectivity in a sample of patients diagnosed with poly-drug use disorder (PUD; ICD-10: F19.2; [32]), a population known for decreased attachment security [33]. We expected alterations in the links between networks previously observed to play a role in attachment and social as well as affective processing. These include the default mode network, salience network, sensory network, the limbic system and further subcortical regions (VTA, PAG and Thalamus). Rs-FC of patients was investigated at two measuring points, 3-weeks apart. A two-step approach was chosen to establish an appropriate seed region, which included 1) an explorative ROI-to-ROI analysis phase for data from the first measurement and 2) based on these findings, a confirmatory seed-to-voxel analysis for data from the second measurement. At the behavioral level, correlations between insecure attachment, depression, state anxiety and general psychiatric symptom load were investigated.

## Materials and methods

### Participants

We investigated a total sample of 33 right-handed participants (Age: M = 30y; SD = 8y; Female = 15%) who were all undergoing long-term substance use disorder treatment within a therapeutic community [34], funded by the “Grüner Kreis” society, at the time of the study. All patients were diagnosed with poly-drug use disorder (ICD-10: F19.2) by a licensed psychiatrist according to the International Classification of Diseases version 10 [32] and reported between 4 and 31 years of illicit drug use. Moreover, before treatment, patients were diagnostically assessed regarding comorbid disorders by licensed clinical psychologists and psychiatrists of the Grüner Kreis society. Pre-treatment, the consumption pattern of all participants was characterized by a chaotic use of psychoactive substances, including almost all substance classes (e.g., opioids, tranquilizer, stimulants, alcohol, cannabinoids, nicotine, etc.) with opioids being the primary drug of abuse in all participants. No substances of abuse, apart from nicotine, were used by the participants at any point during the study. This was evidenced by routine urine screenings conducted within the treatment facility.

The recruitment period began on December 19, 2022 and lasted until March 17, 2023. The study was conducted in accordance with the recommendations of the ethics guidelines of the University of Graz. The Ethics Committee of the University of Graz approved the protocol (GZ. 39/21/63 ex 2022/23). All subjects gave their written consent.

### Procedure

The psychometric assessment of the PUD patients was carried out in a therapeutic community center for addictive disorders, run by the “Grüner Kreis” society. All behavioral tests were conducted in the context of group testing sessions. The data collection was part of a larger controlled study, which investigated therapeutic effects of a dance-therapeutic intervention in PUD patients. For the current study, only baseline measures were assessed. Informed consent was obtained from all participants in accordance with the Declaration of Helsinki.

### MRI acquisition

Imaging data were acquired on a 3T Siemens Vida (Siemens Healtheneers, Erlangen, Germany) with a 64-channel head coil. Participants underwent a scanning procedure that involved a seven-minute resting-state session. During this session, participants were instructed to remain still, relax, and let their minds wander while fixating on a cross displayed on the screen. We obtained functional T2 * -weighted echo-planar images using a multiband sequence with the following parameters: a repetition time (TR) of 1400 ms, an echo time (TE) of 30 ms, a field of view of 256 mm, a flip angle of 65 °, multiband-factor of 4, phase encoding direction from anterior to posterior, and 60 axial slices, resulting in isotropic voxels of 2.5 mm³ covering the entire brain. After the resting state, another functional run of 10 volumes was acquired with the same parameters in the opposite phase encoding direction. This run was used in the analysis as a field map.

Additionally, three-dimensional T1-weighted structural images were acquired using an MPRAGE sequence with the following parameters: a repetition time (TR) of 1970 ms, an echo time (TE) of 2.21 ms, a field of view of 256 mm, a flip angle of 8°, an inversion time of 1000 ms and 224 sagittal slices, resulting in isotropic voxels of 0.9 mm³ covering the entire brain.

### Psychometric assessment

#### Adult attachment.

The German short version of the Experiences in Close Relationships-Revised (ECR-RD8; [35]) is an established self-report questionnaire assessing attachment insecurity in relation to anxiety and avoidance behavior. The questionnaire contains eight items rated on a Likert scale from “strongly disagree” (1) to “strongly agree” (7). The short version total-score measuring overall insecure attachment achieved an acceptable reliability with α = .77.

#### Depression.

The Allgemeine Depressions-Skala [General Depression-Scale] (ADS; German version of the Center for Epidemiological Studies Depression Scale; [36]) is a self-assessment instrument used to measure the impact of depressive symptoms within the last week. The questionnaire includes 20 items inquiring about emotional, motivational, cognitive, somatic, as well as motor/interactive complaints. Cronbach’s α was estimated to be.89.

#### Trait anxiety.

Trait anxiety was measured with the trait scale of the State-Trait-Anxiety-Inventory (STAI; [37]). The trait scale of the STAI consist of 10 Items operationalizing individual anxiety dispositions as differences regarding fear-reaction. Internal consistency was assessed to be excellent with α = .90 [37].

#### Severity of psychiatric symptoms.

General psychiatric symptom load was measured via the Brief Symptom Inventory (BSI-53; [38]). The instrument measures symptoms of somatization, depression, anxiety, aggressiveness, phobic anxiety, paranoid thinking and psychoticism via 53 self-rated items. The total scale (Global Severity Index; GSI) has an excellent internal consistency of α = .96.

### Statistical analysis and analysis strategy

#### Psychometric analysis.

SPSS 29 was used for data management, descriptive statistics and inference statistical analyses of the psychometrically assessed measures. To investigate associations between attachment insecurity, psychopathological symptoms and trait anxiety, bivariate Pearson’s correlations were employed. The alpha level was set to p < 0.05.

#### Preprocessing.

Results included in this manuscript come from preprocessing performed using fMRIPrep 23.0.1 [39], which is based on Nipype 1.8.6 [40]. A detailed description of the anatomical and functional data preprocessing can be found in the supplementary (S1 and S2 File).

#### Functional connectivity analysis and analysis strategy.

Employing the CONN toolbox v22a, we performed resting-state functional connectivity analyses for each participant using the provided Regions of Interest (ROI) atlas in step 1 and the seed to voxel function in t2. At the first measuring point we investigated several ROIs which were included based on previous studies investigating associations with the human attachment. Thus, the CONN default ROI selection was reduced to the network atlas, limbic and subcortical atlas areas (overall 61 ROIs). PAG, VTA and LC were included from the publicly available atlas provided by Levinson, et al. [41]. As a cut-off for significant connections a connection threshold of p < .01 and a cluster threshold of a ROI-level p-FDR corrected (multi-voxel pattern analysis (*MVPA*) omnibus test) was used [42]. To ensure robustness of findings we additionally analyzed the data of t1 with spatial pairwise clustering (SPC mass/intensity cluster threshold p-FDR < .05; connection threshold p < .01). Based on the results of the first measurement we planned to include the most important ROIs of the first analysis as seed regions for the second analysis. This step was conducted on the data of the second measurement. Here, a voxel threshold of p < .001 and cluster-size p-FDR corrected threshold: p-FDR < .05 was established.

At both measuring points results were adjusted for the effects of sex, age, education, medication (antipsychotic medication, maintenance medication, anticonvulsants, selective serotonin reuptake inhibitors, tranquilizer) and general psychopathology as measured by the BSI-53 total score (GSI) by adding corresponding contrasts in CONN. Furthermore, analysis of rs-FC data of the second measurement point was additionally controlled for cofounding effects of the dance-therapeutic intervention.

## Results

### Descriptive statistics

As detailed in Table 1, 42% of participants reported a compulsory school diploma as their highest completed education, while the vast majority (97%) were Austrian citizens. Over half of the participants (52%) did have a psychiatric comorbidity. Furthermore, 36% of participants were in maintenance treatment at the time of the study, and 33% were receiving antidepressant medication.

**Table 1:** Descriptive statistics.

Gender	Female: n = 5
	Male: n = 28
Education	Compulsory school = 14
	Apprenticeship = 13
	High school degree = 6
Nationality	Austria = 32
	Other = 1
Diagnosis	F19.2 = 33
Comorbidity	No comorbidity = 16
	F20 = 3
	F25 = 1
	F32.0 = 3
	F33.1 = 1
	F33.2 = 1
	F41.2 = 1
	F43.1 = 1
	F43.2 = 2
	F50.8 = 1
	F60.3 = 1
	F61 = 4
	F90.0 = 3
Medication	Antipsychotic = 6
	Antidepressant = 11
	Anticonvulsiva = 5
	Tranquilizer = 5
	Maintenance = 12

### Correlation analysis

All investigated variables were tested for non-normality. Kolmogorov-Smirnoff Tests indicated that all variables were normally distributed (p > .05) with the exception of sex (p < .05). Hence, correlations regarding sex were analyzed via spearman-rank-correlations.

Increased insecure attachment was significantly correlated with more symptoms of depression (r = .44; p < .05), higher trait anxiety (r = .44; p < .05), and more general psychiatric symptom burden (r = .51; p < .01). What is more, older patients reported significantly lower insecure attachment (r = −.51; p < .01; see Table 2). Scatterplots for the investigated relationships can be seen in the supplement file S2 File (Figs S1–S3).

**Table 2:** Means, standard deviations and correlations between attachment and psychopathological symptoms.

Variable	1	2	3	4	5	6
1. ECR-Total	**–**					
2. Depression	.44^* ^	–				
3. STAI-Trait	.44^* ^	.87^**^	–			
4. GSI	.51^**^	.85^**^	.79^**^	–		
5. Age	−.51^**^	−.11	−.13	−.28	–	
6. Sex	−.25	−.20	−.16	−.20^* ^	.27	–
*Mean or Frequency*	22.88	18.52	2.18	6.95	30.70	5
*Standard Deviation or %*	8.34	10.03	0.60	5.72	8.09	15.20

**p < .01; *p < .05

### Functional connectivity

For both measuring points the effect of insecure attachment was controlled for the following set of covariates of no interest: Sex, age, education, tranquilizer and general psychopathological symptoms (GSI), antipsychotic medication, maintenance medication, anticonvulsiva, selective serotonin reuptake inhibitors, and tranquilizer medication.

### First measurement (ROI-to-ROI)

**Fig 1:**
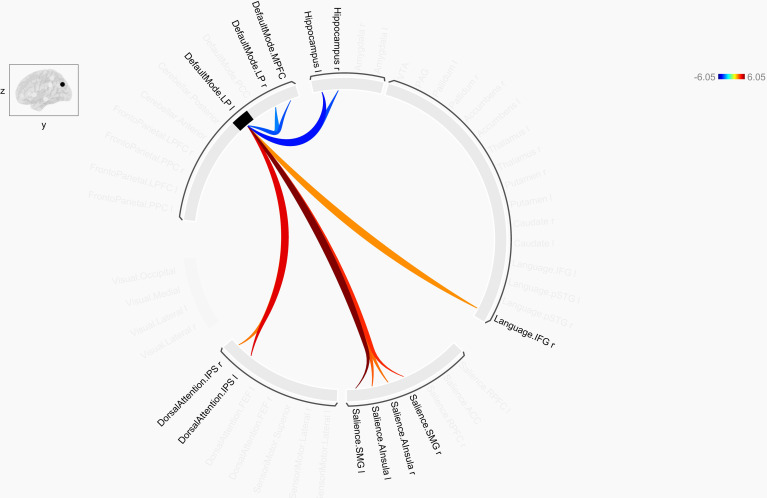
ROI-to-ROI connectivity effects of insecure adult attachment (p-FDR corrected MVPA omnibus test).

As detailed in Table 3, insecure attachment was significantly associated with alterations affecting rs-FC of the left DMN LP. They included increased connectivity with bilateral areas in the salience network, and dorsal attention network intraparietal sulcus, language network inferior frontal gyrus and paracingulate gyrus. Furthermore, decreased rs-FC was observed with the bilateral hippocampus, DMN medial prefrontal cortex (MPFC), and right DMN LP (see Fig 1). Additional analysis with SPC (p-FDR < .05) revealed a similar pattern. As shown in S1 File two significant clusters were observed, with cluster 1 indicating relationships between the bilateral DMN LP with the salience network. The second cluster indicated increased connectivity between the bilateral dorsal attention network with the bilateral hippocampus and amygdala.

**Table 3:** ROI-to-ROI connectivity effects of insecure adult attachment (p-FDR corrected MVPA omnibus test).

MNI Coordinates (x, y, z)	Seed-region	F(2,21)	Targets	t(22)	p-uncorrected	p-FDR
−39, −77, +33	DMN LP (L)	13.14			.000199	.012140
			Salience SMG (L)	6.05	.000004	.000258
			Dorsal Attention IPS (L)	4.89	.000069	.002067
			Hippocampus (L)	−4.36	.000252	.005040
			Salience SMG (R)	4.05	.000533	.007991
			Hippocampus (R)	−3.69	.001279	.013856
			DMN MPFC	−3.66	.001386	.013856
			Salience Insula (L)	3.10	.001951	.015256
			Paracingulate Gyrus (L)	−3.50	.002034	.015256
			Salience Insula (R)	3.25	.003648	.024320
			Dorsal Attention IPS (R)	3.12	.005028	.030166
			Paracingulate Gyrus (R)	−3.07	.005670	.030925
			DMN LP (R)	−3.02	.006356	.031778
			Language IFG (R)	2.85	.009284	.042850

The effect of insecure attachment was controlled for sex, age, education, tranquilizer and general psychopathological symptoms (GSI), antipsychotic medication, maintenance medication, anticonvulsiva, selective serotonin reuptake inhibitors, and tranquilizer; DMN LP = default mode network lateral parietal; SMG = supramarginal gyrus; IPS = intraparietal sulcus; IFG = inferior frontal gyrus; (R) = right hemisphere; (L) = left hemisphere; n = 33; connection threshold: p < .01; cluster threshold: ROI-level p-FDR corrected (MVPA omnibus test) p < .05.

**Fig 2:**
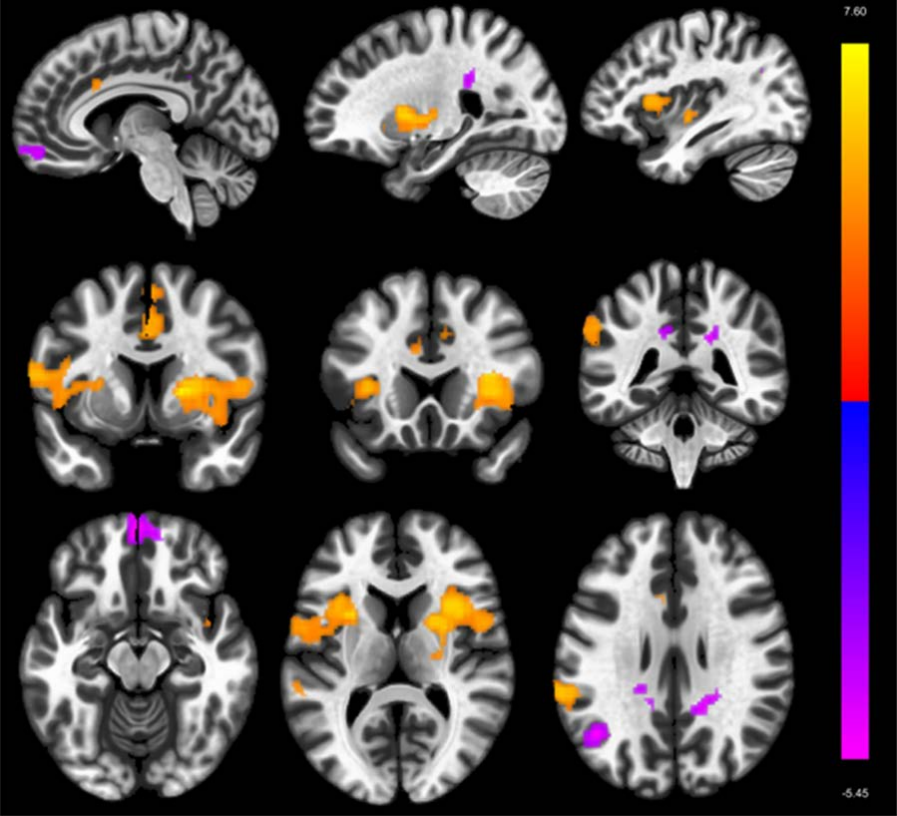
Seed-to-voxel connectivity effects of insecure adult attachment with the left DMN LP as Seed (t2). Colors indicate significant (voxel: p < .001; cluster: p-FDR < .05) effects while grey areas signal non-significant effects.

### Second measurement (Seed to Voxel)

Based on these initial findings the bilateral DMN LP and the bilateral dorsal attention IPS and FEF were chosen as the seed regions of the seed to voxel analysis. As visualized in Fig 2 and detailed in Table 4, results for the left DMN LP indicated 4 clusters comprising – amongst others – parts of the bilateral insula, supramarginal gyrus (SMG) and ACC, which showed increased rs-FC with the DMN LP (t(21) = 7.51 – 5.85; Cluster Voxel Size = 1803 – 464; Voxel p < .001; Cluster p-FDR < .01). Furthermore, 5 negatively correlated clusters (t(21) = −6.45 – −5.04; Cluster Voxel Size = 305 – 124; Voxel p < .001; Cluster p-FDR < .01) were detected which affected parts of the bilateral frontal pole and MPFC, left angular gyrus, right temporal pole, as well as bilateral posterior cingulate cortex.

**Table 4.** Seed-to-Voxel Connectivity effects of insecure adult attachment with left DMN LP as Seed (t2).

MNI Cluster coordinates (x, y, z)	Cluster size (Voxel)	Region	MNI- coordinates of peak voxel (x, y, z)	Voxel (>10)	t(21)	p-FDR
+18, + 02, + 06	1803				7.51	.000002
		Insular Cortex (R)	+38, + 10, + 0	449		
		Putamen (R)	+26, + 2, + 2	416		
		Frontal Operculum Cortex (R)	+40, + 16, + 6	188		
		Central Opercular Cortex (R)	+46, + 6, + 6	130		
		Pallidum (R)	+20, −2, + 0	102		
		Inferior Frontal Gyrus (R)	+52, +10, + 6	41		
		Precentral Gyrus (R)	+54, +6, + 8	31		
		Planum Polare (R)	+52, −4, −2	25		
		Unlabeld	+32, +8, + 6	401		
−26, + 10, + 08	1287				6.93	.000002
		Insular Cortex (L)	−38, +4, + 4	304		
		Central Opercular Cortex (L)	−48, +0, + 6	263		
		Precentral Gyrus (L)	−56, +4, +14	237		
		Frontal Operculum Cortex (L)	−38, +14, +6	148		
		Putamen (L)	−24, +10, +2	105		
		Planum Polare (L)	−48, −6, −2	53		
		Inferior Frontal Gyrus (L)	−48, +8, +14	15		
		Unlabeled	−48, +8, +14	152		
-68,-32, + 24	979				7.16	.000002
		Supramarginal Gyrus, anterior division (L)	−62, −34, +32	301		
		Parietal Operculum Cortex (L)	−54, −34, +20	239		
		Planum Temporale (L)	−56, −34, +16	155		
		Supramarginal Gyrus, posterior division (L)	−60, −44, +32	96		
		Postcentral Gyrus (L)	−62, −20, +20	32		
		Central Opercular Cortex (L)	−58, −20, +16	32		
		Unlabeled	−68, −32, +22	116		
+02, +02, +36	464				5.85	.000013
		Cingulate Gyrus, anterior division	+2, +4, +38	290		
		Supplementary Motor Cortex (R)	+4, +4, +56	91		
		Paracingulate Gyrus (R)	+4, +12, +42	25		
		Supplementary Motor Cortex (L)	−2, +6, +56	11		
		Unlabeled	+0, +6, +52	42		
+02, +58, −14	305				−5.15	.000055
		Frontal Pole (L)	−4, +60, −12	85		
		Frontal Medial Cortex	+0, +52, −12	78		
		Frontal Pole R(R)	+4, +60, −12	77		
		Unlabeled	+8, +58, −14	65		
−44, −60, +32	153				−5.04	.000055
		Angular Gyrus (L)	−44, −58, +30	65		
		Lateral Occipital Cortex (L)	−46, −64, +30	65		
		Unlabeled	−38, −60, +30	23		
+42, +24, −32	133				−5.05	.000055
		Temporal Pole Right	+38, +24, −34	112		
		Unlabeled	+40, +26, −38	21		
+18, 42, +28	128				−6.45	.000004
		Cingulate Gyrus, posterior division (R)	+12, −46, +30	11		
		Unlabeled	+12, −42, +30	117		
−22, −38, +34	124				−6.41	.000004
		Cingulate Gyrus, posterior division (L)	−10, −44, +34	31		
		Unlabeled	−18, −38, +32	93		

The effect of insecure attachment was controlled for sex, age, education, tranquilizer and general psychopathological symptoms (GSI), antipsychotic medication, maintenance medication, anticonvulsiva, selective serotonin reuptake inhibitors, and tranquilizer; DMN LP = default mode network lateral parietal; SMG = supramarginal gyrus; (R) = right hemisphere; (L) = left hemisphere; n = 33.

The right DMN LP showed higher connectivity to one cluster in the left hemisphere (t(21) = 7.11; Cluster Voxel Size = 984; Voxel p < .001; Cluster p-FDR < .01). As detailed in Table 5 this comprised parts of the central opercular cortex, precentral gyrus, planum temporale and insular cortex.

**Table 5:** Seed-to-Voxel connectivity effects of insecure adult attachment with right DMN LP as Seed (t2).

MNI Cluster coordinates (x, y, z)	Cluster size (Voxel)	Region	MNI coordinates of peak voxel (x, y, z)	Voxel (>10)	T(21)	p-FDR
−48, −06, +04	984				7.11	.000001
		Central Opercular Cortex (L)	+50, +10, +10	379		
		Precentral Gyrus (L)	+60, +0, +12	121		
		Planum Temporale (L)	+60, +30, +14	103		
		Insular Cortex (L)	+40, +10, +6	101		
		Planum Polare (L)	+48, +8, +0	58		
		Parietal Operculum Cortex (L)	+58, +30, +16	54		
		Heschl’s Gyrus (L)	+46, +16, +4	52		
		Postcentral Gyrus (L)	+62, +8, +12	11		
		Not Labeled	+64, +8, +8	91		

The effect of insecure attachment was controlled for sex, age, education, tranquilizer and general psychopathological symptoms (GSI), antipsychotic medication, maintenance medication, anticonvulsiva, selective serotonin reuptake inhibitors, and tranquilizer; DMN LP = default mode network lateral parietal, n = 33.

For the dorsal attention network seeds, no significant effects were observed for the second measuring point.

Overall, both measuring points highlighted associations of insecure attachment with increased rs-FC between the DMN LP and the salience network, as well as decreased rs-FC to medial parts of the DMN with the left DMN LP.

## Discussion

In this study, it was intended to explore the relationship between insecure attachment and rs-FC in individuals diagnosed with PUD. Our results indicate associations between insecure attachment and alterations in the rs-FC of the bilateral DMN LP. Namely we observed increased rs-FC involving bilateral salience attention network, as well as reduced connectivity with the hippocampus and within the DMN for the left DMN LP and increased connectivity between the right DMN LP and parts of the left salience network. These findings are paralleled by results suggesting more severe comorbid symptoms in insecurely attached patients.

The DMN LP area corresponds to Brodmann area 39 [43], also known as the angular gyrus (AG). Structurally, the AG serves as a major hub enabling connections between various regions including temporofrontal subsystems and medial regions such as the hippocampus, caudate, and precuneus [44,45]. This area was shown to play an important role in an exceptionally wide variety of functions, spanning from word processing [46] to out-of-body experiences [47]. Meta-analytic evidence implicates the AG consistently in tasks related to theory-of-mind and social cognition [48–50], episodic memory retrieval [51–53], reading comprehension, as well as number and semantic processing [54–56]. Within the DMN, the AG is hypothesized to act as a dynamic self-referential region linked to interoception and integration of somatosensory information, contributing to the construction of mental scenes based on acquired knowledge [57]. This might be related to its involvement in theory-of-mind, mentalization and social cognition, where it supports access to mental representations based on contextual information [50]. As a unified framework for these diverse sets of functions, Seghier [57] proposed the AG as an integrative intersection between bottom-up sensory inputs and DMN mediated top-down predictions [58].

In general, the DMN is comprised of the dorsal medial prefrontal cortex, posterior cingulate cortex, precuneus and angular gyrus and is active during so-called “stimulus-independent thought” or “mind wandering” [43]. During periods of rest, the DMN suppresses other large scale networks like the salience or dorsal attention network [59]. Hypoconnectivity within the DMN was previously linked to a variety of psychopathological phenomena associated with aberrant affect regulation like PTSD [60,61], schizophrenia [62,63] and autism [64].

The hippocampus plays an important role in learning and memory and affect regulation [65]. Hence, both observations of a hypoconnectivity of the DMN LP with other areas of the DMN and the hippocampus in insecurely attached patients might relate to difficulties regarding processing of memory, affect regulation and self-referential information.

Considering areas exhibiting hyperconnectivity, our results particularly highlight alterations between the DMN LP and the salience network. The salience network was shown to be functionally linked to the identification of homeostatically relevant internal and external stimuli and to be responsible for inter-network interactions, working as a “dynamic switch” between other networks [66]. Structural impairment in the salience network usually results in the loss of social-emotional capacities and self-awareness [67]. Within the salience network, the most pronounced and time-stable alterations were found with the insula and the SMG. The insula is situated deep inside the Sylvian fissure, and reciprocally connected to large parts of the limbic system including the amygdala, ventral striatum, as well as the association cortex [68]. It receives afferents from the sensory thalamic nuclei and is involved in a wide variety of functions including pain perception and affective as well as social processing [68,69]. The SMG, corresponding to the Brodmann area 40, is part of the somatosensory and mirror-neuron system [70,71] and was previously associated with language processing, sensorimotor integration and social cognition/empathy [71].

The imbalance between the DMN LP, salience network, and DMN PCC and MPFC may reflect heightened sensitivity to internal and external stimuli [72], increased difficulties in disengaging from these stimuli during resting periods [73], as well as in integrating affective, sensorimotor and reward-related stimuli in general [59,74,75]. In sum, this might translate to difficulties of the DMN LP to regulate and integrate these systems [67,76,77], and may explain some of the severe problems regarding the ability to navigate social and emotional situations effectively connected to insecure attachment.

This interpretation is further supported by the psychometric results indicating moderate to strong associations between comorbid symptoms of depression and general psychopathology and insecure attachment in participants. This reaffirms a substantial body of previous evidence on the association between psychopathology and adult attachment [13,78,79]. Along these lines, previous studies observed similar increases in connectivity between salience network and DMN regarding other psychiatric disorders [80,81].

The findings also align with observations of increased hypervigilance in insecure attached individuals [82–84], and resonate with Carhart-Harris’s and Friston’s [85] conceptualization of the DMN as biological substrate necessary to bind error signals of subordinate-affective brain networks.

Of note, the current study parallels previous findings indicating lowered fractional anisotropy in a segment comprised of parts of the right uncinated fasciculus, inferior fronto-occipital fasciculus and anterior thalamic radiation in more avoidant PUD patients [86] and healthy individuals [28]. Because the identified white matter fiber tracts are centred on connections of the right insula cortex [87], these results potentially underscore the role of this particular structure in adult attachment patterns. However, more work will be needed to further investigate the relationship between structural and functional connectivity in this regard.

### Limitations and future perspectives

Our results show considerable divergence from previous studies exploring links between rs-FC and adult attachment. While both Rigon et al. [28] and van Hoof et al. [30] observed changes in connectivity involving overlapping parts of the DMN as observed in the present study, we did not find significant variations in connectivity of the amygdala in correspondence to increased attachment insecurity. The particular population investigated may explain part of the divergence, as both Rigon et al. as well as van Hoof et al. based their analysis on healthy samples. Given previous observations which demonstrated serious alterations concerning structural connectivity in PUD patients [88–90], generalizability to the general population of our rs-FC results might be specifically limited. Moreover, in contrast to Rigon et al. [28] and Deng et al. [29], we did not investigate avoidant and anxious attachment styles separately but used a total score of the ECR-RD which measures general attachment insecurity, corresponding to the fearful-avoidant style proposed by Bartholomew and Horowitz (8). Regarding patients suffering from severe forms of PUD, this conflicted style has strong clinical significance [1,91–93].

While the ECR-RD8 exhibits several psychometric advantages [35], and due to its briefness is especially suited for the investigation of severely impacted patients, further research will be needed employing the long form of this instrument. Specifically, the relatively low (but still acceptable) reliability the total scale might be improved by this procedure. Moreover, future studies should also explore the effects of both anxious and avoidant attachment dimensions separately. Similarly, differences between separate clusters of attachment styles might be interesting to study regarding their rs-FC. However, as of now, the ECR-RD8 does not provide representative norm values, and thus, attachment security had to be investigated as a continuum in the present study.

An additional consideration is the potential impact of drug use duration onto rs-FC of salience and affect regulating networks, which is highlighted by recent meta-analytic evidence [94]. In our study, drug use duration was assessed in a relatively limited way, which may not fully capture the complex drug-use histories common among patients with PUD. As a result, we did not include it as an additional covariate. Future studies, however, would benefit from a more detailed assessment of drug use duration to better estimate and control for this confounding variable.

Notably, the investigated patients were undergoing an inpatient treatment within a therapeutic community at the time of the study. This specific therapeutic approach is based on ideas of collective responsibility, participation and empowerment [95] and is therefore, distinguished by a high degree of social cohesion. Consequently, this might have influenced the observed results. Furthermore, despite its inclusion as covariate of no interest the dance-therapeutic intervention might have inflated findings regarding motor related areas like the putamen and pallidum in the second measurement. Relating to the specificity of the investigated sample and the relatively small sample size, more research aiming to replicate and further explore these findings will be necessary. Specifically, future studies would benefit from including a matched healthy control group, in order to derive higher generalizability. Based on our current results, it is only possible to show rs-FC correlates of attachment insecurity in PUD patients. In the light of the ongoing discussion regarding the apparent variability of neuroimaging studies [96] the presented results should be seen as a building block for further (meta-) analyses and hence, the suggested interpretation have to remain preliminary at this stage.

Furthermore, the current study employed static rs-FC. Future research should consider conducting supplementary dynamic FC analyses on the neuronal underpinnings of attachment security, as recent evidence indicates alteration in dynamic FC in association to psychopathological phenomena [97]. Finally, the age and gender distribution was uneven, which however, not only reflects the realities of a therapeutic community population, but was also statistically accounted for.

Considering the relative homogenous sample, which was extensively controlled for effects of possible cofounders like sex, age, education, general psychopathology and medication, as well as the two-step measurement approach, the results pose an important step towards the neuroscientific mapping of insecure attachment in PUD patients. These efforts aim to create elementary units for the development of neuroscientifically informed therapeutic approaches and outcome criteria for this notoriously difficult-to-treat and life-threatening disorder.

## Conclusion

In sum, our results strengthen the assumption regarding a connection between attachment insecurity and affect regulation, and therefore, emphasize the necessity for treatment approaches which focus on the improvement of affect regulation mechanisms.

Relevant for therapeutic interventions, research suggests relatively extensive training-induced plasticity in the DMN-LP of the adult brain [98–101], especially in correspondence to acquiring new skills connected to spatial coordination and verbal memory. Hence, future research could explore whether psychotherapeutic interventions focusing on language and motor skills impact attachment security and related rs-FC.

## Supporting information

S1 File Anatomical data preprocessing. (DOCX)

S2 File Functional data preprocessing. (DOCX)
