# Supplementary material for: Effects of insecure attachment on fMRI resting state functional connectivity in poly drug use disorder
Source: PLoS One. 2025 Feb 12;20(2):e0318505. doi: 10.1371/journal.pone.0318505 (PMC11819475; doi:10.1371/journal.pone.0318505)
Supplement: S1 File — (DOCX) [file pone.0318505.s001.docx]

**S1. Anatomical data preprocessing**

The T1-weighted image was corrected for intensity non-uniformity (INU) with N4BiasFieldCorrection (1), distributed with ANTs (2), and used as T1w-reference throughout the workflow. The T1w-reference was then skull-stripped with a Nipype implementation of the antsBrainExtraction.sh workflow (from ANTs), using OASIS30ANTs as the target template. Brain tissue segmentation of cerebrospinal fluid (CSF), white matter (WM), and grey matter (GM) was performed on the brain-extracted T1w using fast (3). Volume-based spatial normalization to one standard space (MNI152NLin2009cAsym) was performed through nonlinear registration with antsRegistration (ANTs), using brain-extracted versions of both T1w reference and the T1w template. The ICBM 152 Nonlinear Asymmetrical template version 2009c (4, 5); emplateFlow ID: MNI152NLin2009cAsym) was used as a T1w template.

**S1. Functional data preprocessing**

First, a reference volume and its skull-stripped version were generated. Head-motion parameters concerning the BOLD reference (transformation matrices and six corresponding rotation and translation parameters) are estimated before spatiotemporal filtering using mcflirt (FSL, (6)). The BOLD run was slice-time corrected to 0.644s (0.5 of slice acquisition range 0s-1.29s) using 3dTshift from AFNI (7). The BOLD time series were resampled onto their original, native space by applying the transforms to correct for head motion. The BOLD reference was then co-registered to the T1w reference using mri_coreg (FreeSurfer) followed by flirt (FSL, (6)) with the boundary-based registration (8) cost-function. Co-registration was configured with six degrees of freedom. Several confounding time series were calculated based on the preprocessed BOLD: DVARS and three region-wise global signals were calculated for each functional run, both using their implementations in Nipype (9). The three global signals are extracted within the CSF, the WM, and the whole-brain masks. Additionally, physiological regressors were extracted for component-based noise correction (CompCor). Principal components are estimated after high-pass filtering the preprocessed BOLD time series (using a discrete cosine filter with 128s cut-off) for the two CompCor variants: temporal (tCompCor) and anatomical (aCompCor). tCompCor components are then calculated from the top 2% variable voxels within the brain mask. For aCompCor, three probabilistic masks (CSF, WM and combined CSF+WM) are generated in anatomical space. Finally, these masks are resampled into BOLD space and binarized by thresholding at 0.99 (as in the original implementation). Components are also calculated separately within the WM and CSF masks. For each CompCor decomposition, the k components with the largest singular values are retained, such that the retained components’ time series are sufficient to explain 50 percent of variance across the nuisance mask (CSF, WM, combined, or temporal). The remaining components are dropped from consideration. The head-motion estimates calculated in the correction step were also placed within the corresponding confounds file. The confound time series derived from head motion estimates and global signals were expanded by including temporal derivatives and quadratic terms for each (10). Frames that exceeded a threshold of 0.5 mm FD or 1.5 standardized DVARS were annotated as motion outliers. Additional nuisance timeseries are calculated employing principal components analysis of the signal found within a thin band (crown) of voxels around the edge of the brain, as proposed by (11). The BOLD time series were resampled into standard space, generating a preprocessed BOLD run in MNI152NLin2009cAsym space. First, a reference volume and its skull-stripped version were generated using a custom methodology of fMRIPrep. All resamplings can be performed with a single interpolation step by composing all the pertinent transformations (i.e., head-motion transform matrices, susceptibility distortion correction when available, and co-registrations to anatomical and output spaces). Gridded (volumetric) resamplings were performed using antsApplyTransforms (ANTs), configured with Lanczos interpolation to minimize the smoothing effects of other kernels (12). Non-gridded (surface) resamplings were performed using mri_vol2surf (FreeSurfer; (13)).

Preprocessed data from fmriprep was then fed into the CONN toolbox ((14); RRID:SCR_009550) release 22a. Functional data were smoothed using spatial convolution with a Gaussian kernel of 6 mm full-width half maximum (FWHM). In addition, functional data were denoised using a standard denoising pipeline (15), including the regression of potential confounding effects characterized by white matter timeseries (5 CompCor noise components), CSF timeseries (5 CompCor noise components), motion parameters and their first order derivatives (12 factors; (16)), outlier scans (below 247 factors; (9)), session and task effects and their first order derivatives (2 factors), and linear trends (2 factors) within each functional run, followed by high-pass frequency filtering of the BOLD timeseries (17) above 0.008 Hz. CompCor (18, 19) noise components within white matter and CSF were estimated by computing the average BOLD signal and the largest principal components orthogonal to the BOLD average, motion parameters, and outlier scans within each subject's eroded segmentation masks. From the number of noise terms included in this denoising strategy, the effective degrees of freedom of the BOLD signal after denoising were estimated to range from 143.7 to 385.2 (average 357) across all subjects (20).

1. Tustison NJ, Avants BB, Cook PA, Zheng Y, Egan A, Yushkevich PA, et al. (2010): N4ITK: improved N3 bias correction. *IEEE Trans Med Imaging*. 29:1310-1320.

2. Avants BB, Epstein CL, Grossman M, Gee JC (2008): Symmetric diffeomorphic image registration with cross-correlation: evaluating automated labeling of elderly and neurodegenerative brain. *Med Image Anal*. 12:26-41.

3. Zhang Y, Brady M, Smith S (2001): Segmentation of brain MR images through a hidden Markov random field model and the expectation-maximization algorithm. *IEEE Trans Med Imaging*. 20:45-57.

4. Ciric R, Thompson WH, Lorenz R, Goncalves M, MacNicol EE, Markiewicz CJ, et al. (2022): TemplateFlow: FAIR-sharing of multi-scale, multi-species brain models. *Nat Methods*. 19:1568-1571.

5. Fonov VS, Evans AC, McKinstry RC, Almli CR, Collins D (2009): Unbiased nonlinear average age-appropriate brain templates from birth to adulthood. *NeuroImage*. 47:S102.

6. Jenkinson M, Smith S (2001): A global optimisation method for robust affine registration of brain images. *Med Image Anal*. 5:143-156.

7. Cox RW, Hyde JS (1997): Software tools for analysis and visualization of fMRI data. *NMR Biomed*. 10:171-178.

8. Greve DN, Fischl B (2009): Accurate and robust brain image alignment using boundary-based registration. *Neuroimage*. 48:63-72.

9. Power JD, Mitra A, Laumann TO, Snyder AZ, Schlaggar BL, Petersen SE (2014): Methods to detect, characterize, and remove motion artifact in resting state fMRI. *Neuroimage*. 84:320-341.

10. Satterthwaite TD, Elliott MA, Gerraty RT, Ruparel K, Loughead J, Calkins ME, et al. (2013): An improved framework for confound regression and filtering for control of motion artifact in the preprocessing of resting-state functional connectivity data. *Neuroimage*. 64:240-256.

11. Patriat R, Reynolds RC, Birn RM (2017): An improved model of motion-related signal changes in fMRI. *Neuroimage*. 144:74-82.

12. Lanczos C (1964): Evaluation of Noisy Data. *Journal of the Society for Industrial and Applied Mathematics Series B Numerical Analysis*. 1:76-85.

13. Fischl B (2012): FreeSurfer. *Neuroimage*. 62:774-781.

14. Whitfield-Gabrieli S, Nieto-Castanon A (2012): Conn: a functional connectivity toolbox for correlated and anticorrelated brain networks. *Brain Connect*. 2:125-141.

15. Nieto-Castanon A (2020): *Handbook of functional connectivity magnetic resonance imaging methods in CONN*. Hilbert Press.

16. Friston KJ, Williams S, Howard R, Frackowiak RS, Turner R (1996): Movement-related effects in fMRI time-series. *Magn Reson Med*. 35:346-355.

17. Hallquist MN, Hwang K, Luna B (2013): The nuisance of nuisance regression: spectral misspecification in a common approach to resting-state fMRI preprocessing reintroduces noise and obscures functional connectivity. *Neuroimage*. 82:208-225.

18. Behzadi Y, Restom K, Liau J, Liu TT (2007): A component based noise correction method (CompCor) for BOLD and perfusion based fMRI. *Neuroimage*. 37:90-101.

19. Chai XJ, Castanon AN, Ongur D, Whitfield-Gabrieli S (2012): Anticorrelations in resting state networks without global signal regression. *Neuroimage*. 59:1420-1428.

20. Nieto-Castanon A (2022): Preparing fMRI Data for Statistical Analysis. *arXiv preprint arXiv:221013564*.
